# Effects of Astragalus Polysaccharides Nanoparticles on Cerebral Thrombosis in SD Rats

**DOI:** 10.3389/fbioe.2020.616759

**Published:** 2020-12-23

**Authors:** Qian Sun, Pengqiang Shi, Cuiling Lin, Jing Ma

**Affiliations:** ^1^Department of Neurology, Xinxiang Central Hospital, Xinxiang, China; ^2^Department of Neurosurgery, Xinxiang Central Hospital, Xinxiang, China; ^3^Intensive Care Unit, Xinxiang Central Hospital, Xinxiang, China

**Keywords:** astragalus polysaccharide, nanoparticles, cerebral thrombosis, brain edema, hemorheology, blood coagulation function

## Abstract

**Objective:**

To investigate the efficacy and improvement of Astragalus polysaccharides (APS) and APS-nano on cerebral thrombosis in rats.

**Methods:**

A total of 72 SD rats were randomly divided into NC group, Model group, APS-Nano group, and APS group. The cerebral thrombosis Model of SD rats was established by injecting compound thrombus inducer into the internal carotid artery. After 14 days of different intervention treatments, the TTC staining of brain tissue were performed, and A/left brain wet weight ratio, left brain/right brain wet weight ratio, blood rheology indexes, and coagulation function indexes of cerebral thrombosis were measured. ELISA was used to measure the contents of thromboxane 2 (TXB2), 6-keto-prostaglandin F1α (6-Keto-PGF1α), tissue factor (TF), neuron-specific enolase (NSE), S-100β, catenin (CAT), superoxide dismutase (SOD), as well as malondialdehyde (MDA). The binding specificity between miR-885-3p and TF was verified by the double-luciferin reporting experiment, and western blot was used to measure the expression level of TF protein.

**Results:**

Compared with the Model group, after treatment with APS-nano or APS, the ratio of left brain/right brain wet weight decreased significantly. Whole blood low shear viscosity (WBLSV), whole blood high shear viscosity (WBHSV), plasma viscosity (PV), and erythrocyte aggregation index (Arbc) was all reduced. In addition, prothrombin time (PT) and activated partial thromboplastin time (APTT) were increased, and fibrinogen (FIB) content was decreased. The expression of TXB2, 6-Keto-PGF1α, and TF showed a downward trend. Similarly, the expression of TF protein was decreased. Furthermore, the contents of NSE and S-100β proteins were all decreased, whereas the contents of CAT and SOD were increased, and the contents of MDA was decreased. At the same dose, compared with APS treatment, APS-nano treatment had a significant inhibitory effect on cerebral thrombosis in rats. Finally, we found that TF is a target gene of miR-885-3p and specifically binds to miR-885-3p.

**Conclusion:**

APS has a significant inhibitory effect on the formation of cerebral thrombosis induced by compound thrombus inducers. Moreover, APS-nano has a more significant inhibitory effect on cerebral thrombosis. Meanwhile, the regulation of miR-885-3p regulating TF expression may be related to the occurrence of cerebral thrombosis.

## Introduction

Cerebral thrombosis, a type of stroke is a common type of acute neurological disease. The incidence rate of cerebral thrombosis has been increased recently. Cerebral thrombosis is formed by abnormal blood flow, abnormal blood composition, and damage to blood vessel walls, and has a poor prognosis and many sequelae (Ding et al., 2017). When blood vessel stenosis occurs, it will lead to blood vessel occlusion, thickening, and thrombosis. In patients with cerebral thrombosis, local blood flow is reduced, supply is interrupted, which can lead to cerebral ischemia, hypoxia, softening and necrosis (Mehta et al., 2019). “Shennong’s Classic of Materia Medica” records that astragalus has the effects of stagnation and diuresis, and it is a commonly used medicinal material in clinical trials of traditional Chinese medicine. Modern pharmacological studies have shown that astragalus can regulate cardiovascular activity, and could exert anti-platelet aggregation, improve blood rheology, protect blood vessels, resist fibrosis, and other pharmacological effects (Fu et al., 2014). In the research on the effective ingredients of Chinese medicine, it is found that most of the effective ingredients of tonic Chinese medicines exist in the form of polysaccharides, Astragalus Polysaccharides (APS) is one of the main effective ingredients of Astragalus which not only can effectively improve the microcirculation of patients with cerebral thrombosis but also effectively eliminate free radicals, improve the fluidity of red blood cell and the activity of superoxide dismutase (Wu, 1989), that has been widely used in the clinical treatment of cardiovascular diseases. The blood-brain barrier is a dynamic interface between blood and brain tissue that selectively prevents substances from passing through. Therefore, it can effectively prevent harmful substances from damaging the brain tissue, but at the same time it also prevents many valuable biological drugs from entering the brain lesion area and thus weakening the therapeutical effectiveness. Nanoparticles are characterized by their small size and can cross the brain-blood barrier. Modern pharmacological research showed that the application of nanotechnology can improve the activity and bioavailability of traditional Chinese medicine, and even produce new properties (Chen et al., 2020), therefore, it is expected to become effective drug carriers. MicroRNA (miRNA) is a type of non-coding single-stranded RNA with a length of about 22 nucleotides that regulates the expression of its target gene essentially by translation inhibition or degradation of corresponding transcripts. It has been reported that a variety of miRNAs play a role in thrombosis, such as miR-150 (He et al., 2016), miR-126 (Wang et al., 2018), miR-200b (Kim et al., 2016), miR-495 (Kim et al., 2016), and so on. Among them, miR-885-3p is involved in blood system development, cell death, nervous system development, etc (Hunsberger et al., 2012). In this study, a cerebral thrombosis model rat was established by injecting a compound thrombus inducer into the internal carotid artery to explore the protective effect of astragalus polysaccharide nanoparticles on the brain tissue of cerebral thrombosis model rats and its improvement of blood rheology index and coagulation function, and finally provide some experimental basis for the clinical treatments of cerebral thrombosis.

## Materials and Methods

### Drugs and Reagents

Astragalus polysaccharide (99% purity), was provided by Nanjing Zelang Pharmaceutical Technology Co., Ltd. Polylactic acid-polyethylene glycol copolymer (PEG-PLA), dichloromethane, polyvinyl alcohol, sulfuric acid, phenol, acetone, sodium sulfate, and distilled water were purchased from Jinan Daigang Biotechnology Co., Ltd. TTC staining kit was purchased from Shanghai Kanglang Biotechnology Co., Ltd. Human umbilical vein endothelial cell lines (HUVECs) were purchased from the Wuhan University Typical Culture Collection Center. Elisa test kits were purchased from Beijing Dakco Biotechnology Co., Ltd. TF-WT and TF-MUT were designed and synthesized by Qingke Biological Co., Ltd. miR-885-3p mimics and NC-mimics were designed and synthesized by Shanghai Jima Pharmaceutical Company.

### Preparation of Astragalus Polysaccharide Nanoparticles

First, the astragalus polysaccharide solution was added to the PEG-PLA dichloromethane solution to obtain a milky white emulsion under high-speed stirring. After that, the emulsion was added to the polyvinyl alcohol aqueous solution and stirred at high speed for 3 min, and the double emulsion was stirred with a magnetic stirrer at the speed of 200 rpm until the organic solvent is completely volatilized. Thereafter, the remaining emulsion was centrifuged for 15 min at high speed, the supernatant was discarded, and the precipitate was taken. Finally, the precipitate was washed three times with distilled water and freeze-dried to obtain astragalus polysaccharide nanoparticles.

### Detection of Astragalus Polysaccharide Nanoparticles

#### Making a Standard Curve

100 mg Astragalus polysaccharide nanoparticles were dissolved with water in a 100 mL volumetric flask to obtain the astragalus polysaccharide nanoparticle solution. To get the gradient concentration of astragalus polysaccharide nanoparticle solution, 2 mL of the prepared astragalus polysaccharide nanoparticle solution was drawn in a test tube, and subsequently 1 mL of 5% phenol solution was added. After mixing, 5 mL of concentrated sulfuric acid was added. After vigorous shaking, the solution was heated for 15 min with boiling water and cooled with ice-water mixture. Finally, phenol and sulfuric acid solution were used as blank control, and the absorbance was measure at 491 nm.

#### Stability Determination

The same batch of astragalus polysaccharide nanoparticles was dissolved in dichloromethane. After dissolving, 5 mL of distilled water was added and thoroughly mixed. After standing for 1 h, the aqueous phase was collected. Repeating the above steps for the remaining oil phase, which was first centrifuged at 2,000 rpm for 5 min, and then 2 mL of supernatant was added in a test tube. After that, 1 mL of 5% phenol solution was added, mixed and 5 mL of concentrated sulfuric acid was added. Thereafter, the solution was mixed and heated in boiling water for 15 min. Finally, the nanoparticle stability testing was conducted at a wavelength of 491 nm.

#### *In vitro* Release Test

An appropriate amount of astragalus polysaccharide nanoparticles was weighed and dissolved in 50 mL of normal saline. Then, 2 mL of astragalus polysaccharide nanoparticle solution was taken at 0, 1, 2, 3, 4, 6, and 10 days, respectively, to perform the stability test according to the operation in the stability testing. The ultraviolet-visible light photometer was used to measure the absorbance of astragalus polysaccharide released into physiological saline at different times, and the same batch of astragalus polysaccharide nanoparticles after ultrasonication was used to determine the absorbance with the same method to calculate the cumulative release rate at different times.

### Cell Culture and Transfection

Human umbilical vein endothelial cell lines were cultured in RPMI-1640 medium containing 10% FBS and double antibodies at 37°C and 5% CO_2_. The fluid was exchanged every 3 days. HUVECs in the logarithmic growth phase were inoculated into 6-well plates with approximately 2 × 10^5^ cells per well. Lipofectamine 2000 liposome transfection reagent with stable transfection efficiency was used for transfection. According to the experimental design, miR-885-3p mimics and miR-NC mimics (NC-mimics) were transferred into HUVECs, meanwhile, a blank control was used, and the same amount of liposome transfection reagent was added to it. After the transfection, the cells were cultured for 72 h under culture conditions.

### Construction of Animal Models of Cerebral Thrombosis and Animal Grouping

Seventy two SD rats weighing about 200∼250 g were purchased. These rats were randomly divided into four groups, 18 rats in each group, namely NC group, Model group, APS-nano group (200 mg/kg d), APS group (200 mg/kg d). Except for the injection of normal saline in the NC group, the rats in the other groups were injected with a compound thrombosis inducer (1.25 mmol/L ADP solution, 125,000 units/L thrombin solution, 1 g/L epinephrine) into their common carotid artery, 0.1 mL of compound thrombosis inducer was injected per 100 g of rats. The clip was open during injection and closed again after injection to form an experimental thrombus model. Gavage once a day for 14 days. Then, subsequent experimental operations were carried out.

### TTC Staining of Brain Tissue of SD Rats With Cerebral Thrombosis

Two SD rats in each group were randomly selected for intraperitoneal injection anesthesia, they were quickly decapitated on ice, and the brain tissue was carefully removed. The brain tissue was quick-frozen in the refrigerator at −20°C for 20 min and sliced with 2 mm intervals. The slices were placed in a 2% TTC staining solution for 30 min, and the container was rotated slightly at intervals of 5 min to fully stain. After staining, the sections were washed three times with 1 × PBS, fixed with 10% paraformaldehyde for 6 h, and then photographed.

### Detection of the Degree of Thrombosis and the Severity of Cerebral Edema

Eight SD rats of each group were randomly selected and injected with 0.5% Evans blue solution. After 10 min of treatment, they were quickly decapitated on ice and the brain was removed, washed with physiological saline, and the remaining water was absorbed with filter paper. Then, the brain was weighed and cut. After that, the reagent (0.5% Na_2_SO_4_: acetone = 3: 7) was added and the brain tissue was ground to form a homogenate (add 5 mL of solution per gram of brain tissue), which stood at 4°C for 5 h and centrifuged at 4,000 rpm for 10 min to collect the supernatant and centrifuge again. Supernatant was taken, and the normal saline was used as the standard, the absorbance (A) value at 620 nm was measured by using a spectrophotometer. The ratio of the absorbance value of the left infarct area to the left-brain wet weight represents the content of Evans blue, which is the severity of cerebral thrombosis. The ratio of left-brain wet weight to right-brain wet weight indicates the severity of cerebral edema in rats.

### Determination of Blood Rheology and Coagulation Function Related Indicators

The remaining 8 SD rats in the remaining groups were anesthetized by intraperitoneal injection, and blood was collected from the abdominal aorta. Then, the blood rheology indicators were detected using an automatic blood rheology tester, including whole blood low shear viscosity (WBLSV), Whole blood high shear viscosity (WBHSV), Plasma viscosity (PV), and Red cell assembling index (Arbc). Also, an automatic coagulation analyzer was used to determine the thrombin time (TT), prothrombin time (PT), activated partial thromboplastin time (APTT), and fibrinogen (FIB) content.

### Elisa Detection

The blood sample obtained above was used and centrifuged at 4,000 rpm for 30 min at 4°C. After that, the serum was taken, and the expression of thromboxane B2 (TXB2), 6-keto-prostaglandin F1α (6-Keto-PGF1α), and tissue factor (TF) was measured using the Elisa test kit. In addition, the contents of neuron-specific enolase (NSE), S-100β, catenin (CAT), superoxide dismutase (SOD), as well as Malondialdehyde (MDA) were measured.

### Dual-Luciferase Report Experiment

HUVECs cells were prepared as a single cell suspension, seeded in 96-well plates, and co-transfected with liposome transfection reagent Lipofectamine 2000 according to the instructions. After that, the cells were divided into the following four groups: (1) wild-type plasmid control group (Transfection of NC mimics and TF-WT); (2) Wild-type experimental group (transfection of miR-885-3p mimics and TF-WT); (3) Mutant plasmid control group (transfection of mimics-NC and TF-MUT); (4) Mutant plasmid experimental group (transfected miR-885-3p mimics and TF-MUT). After culturing them for 24 h, the cells were lysed, and the luciferase activity was detected according to the instructions of the dual-luciferase activity detection kit. Relative luciferase activity = firefly luciferase activity value/Renilla luciferase activity value.

### Western Blot

Cells of rat brain tissue of each group were separated to prepare a cell suspension, centrifuged at 1,000 rpm for 5 min, and the supernatant was discarded. Then, the brain tissue was washed twice with 1×PBS and centrifuged at the same conditions as above. After that, an appropriate amount of cell lysate (containing protease inhibitors) was added to lysis for 10 min, centrifuged at 12,000 rpm for 5 min, and the supernatant was used to measure the protein concentration using the BCA method. Then, an appropriate amount of protein was used for SDS-PAGE electrophoresis, wet transferred to PDVF membrane, treated with 5% skimmed milk powder, blocked at 4°C overnight and the membrane was washed three times with 1×PBST (containing 0.05% Tween-20). Thereafter, the corresponding primary antibody (1:1000) was added and incubated overnight at 4°C in a shaker. After washing, the secondary antibody (1:10000) was added and incubated at room temperature for 1 h. Again, after washing, the ECL developing solution was added to the gel imager for imaging.

### Statistical Methods

GraphPad Prism 6 software was used for statistical data analysis, expressed as mean ± SD (mean ± SD), single-factor analysis of variance (ANOVA) was used to compare the differences between multiple sets of data, and *t*-test was used to compare the difference between two sets of data. *P* < 0.05 indicates statistical significance.

## Results

### Determination of Astragalus Polysaccharide Nanoparticles

**FIGURE 1:**
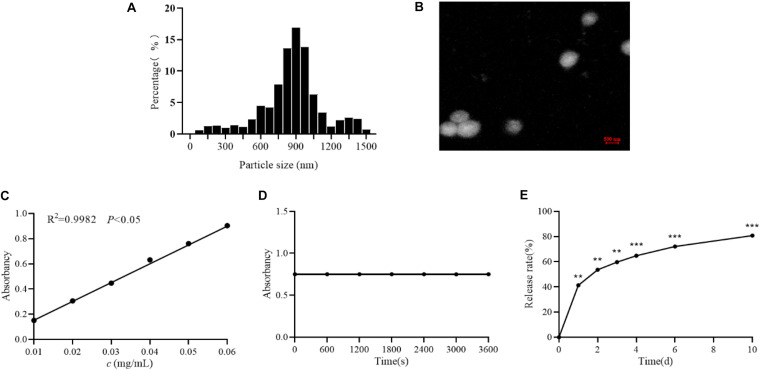
Determination of APS nanoparticles. **(A)** The particle size distribution of Astragalus polysaccharide nanoparticles. **(B)** Transmission electron microscopy of Astragalus polysaccharide nanoparticles. **(C)** Standard curve of Astragalus polysaccharide nanoparticles. **(D)** Stability test of Astragalus polysaccharide nanoparticles. **(E)** The release rate of astragalus polysaccharide nanoparticles *in vitro*. **P < 0.01 and ***P < 0.001, compared with 0 h group.

The spectrophotometer was used to measure the prepared astragalus polysaccharide nanoparticles. The results showed that the particle size of astragalus polysaccharide nanoparticles is mainly distributed between 700 and 1000 nm, with an average particle size of 893 nm, as shown in Figure 1A. And the TEM image was shown in Figure 1B. Besides, astragalus polysaccharide nanoparticles had a good linear relationship in the concentration range of 0.01∼0.06 mg/mL (*R*^2^ = 0.9982; *P* < 0.05), see Figure 1C. The same batch of astragalus polysaccharide nanoparticles was used for stability tests at a wavelength of 491 nm. The results showed that astragalus polysaccharide nanoparticles are stable in color development within 60 min and have good stability, see Figure 1D. Subsequently, an *in vitro* release test of astragalus polysaccharides was conducted and the results showed that astragalus polysaccharide nanoparticles release smoothly *in vitro*, and the release time is longer, see Figure 1E.

### Effects of Astragalus Polysaccharides and Nanoparticles on Rats With Cerebral Thrombosis

**FIGURE 2:**
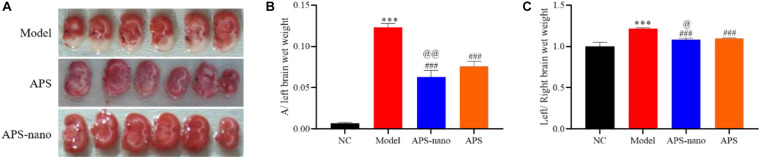
Effects of APS and APS nanoparticles on cerebral thrombosis model rats. **(A)** Cerebral infarction in SD rats with TTC staining. **(B)** Cerebral thrombosis SD rats A/left cerebral wet weight ratio. **(C)** Cerebral thrombosis SD rats left brain/right brain wet weight ratio. ^∗∗∗^*P* < 0.001, compared with NC group; ^###^*P* < 0.001, compared with Model group; ^@^*P* < 0.05 and ^@@^*P* < 0.01, compared with APS group.

TTC staining results showed that compared with the Model group, the white area of the brain tissue of the APS group was significantly reduced (*P* < 0.05), and the white area of the brain tissue of the APS-nano group was also significantly reduced compared to the APS group (*P* < 0.05), as shown in Figure 2A. Compared with the NC group, the ratio of rat A/left brain wet weight in the Model group increased significantly (*P* < 0.001). Compared with Model, the ratio of A/left brain wet weight in the APS-nano group and the APS group was significantly lower (*P* < 0.001). Compared with the APS group, the ratio of A/left brain wet weight in the APS-nano group was significantly lower (*P* < 0.01), as shown in Figure 2B. Compared with the NC group, the left brain/right brain wet weight ratio of rats in the model group was significantly increased (*P* < 0.001). Whereas compared with the Model group, the left brain/right brain wet weight ratio of the rats in the APS-nano group and the APS group decreased significantly (*P* < 0.001). Moreover, compared with the APS group, the left brain/right brain wet weight ratio of the rats in the APS-nano group decreased significantly (*P* < 0.05), as shown in Figure 2C.

### The Effects of Astragalus Polysaccharides and Nanoparticles on Blood Rheology Related Indicators and Coagulation Function in Rats With Cerebral Thrombosis

**FIGURE 3:**
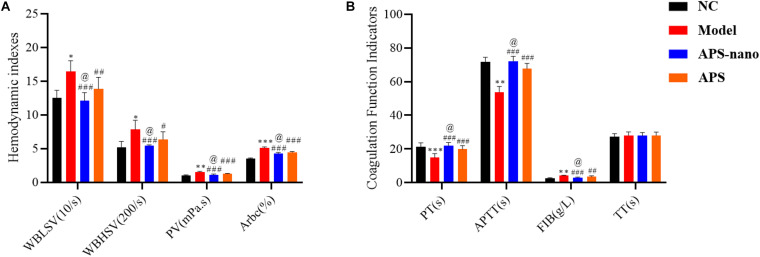
Effects of APS and APS nanoparticles on hemorheology and coagulation function of cerebral thrombosis model rats. **(A)** Changes of hemorheology indexes in cerebral thrombosis model rats. **(B)** Changes of coagulation function in cerebral thrombosis model rats. ^∗^*P* < 0.05, ^∗∗^*P* < 0.01, and ^∗∗∗^*P* < 0.001, compared with NC group; ^#^*P* < 0.05, ^##^*P* < 0.01, and ^###^*P* < 0.001, compared with Model group; ^@^*P* < 0.05, compared with APS group.

As shown in Figure 3A, the WBLSV, WBHSV, PV, and Arbc of rat in the Model group showed a significantly increasing trend (*P* < 0.05) compared to the NC group. However, compared to the Model group, the WBLSV, WBHSV, PV, and Arbc of rats in the APS-nano group and the APS group were reduced to some extent (*P* < 0.05). Furthermore, the WBLSV, WBHSV, PV, and Arbc of rat blood in the APS-nano group were also significantly reduced compared with the APS group (*P* < 0.05). As shown in Figure 3B, the PT and APTT of the Model group were significantly reduced (*P* < 0.01), and the FIB content was increased (*P* < 0.01) compared with the NC group, but the PT and APTT of the rats in both APS-nano group and the APS group were increased (*P* < 0.001), and the FIB content was decreased (*P* < 0.01) compared to the Model group. Similarly, the PT and APTT in the APS-nano group were significantly increased (*P* < 0.05), and the FIB content was significantly reduced (*P* < 0.05) compared with the APS group. There was no obvious change in terms of TT in each group of rats, see Figure 3B.

### Effects of Astragalus Polysaccharides and Nanoparticles on the Expression of TXB2, 6-Keto-PGF1α and TF in Rats With Cerebral Thrombosis

**FIGURE 4:**
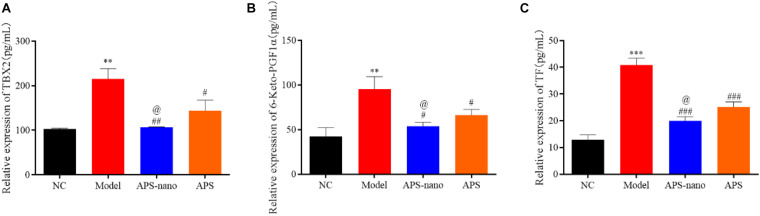
Effects of APS and APS nanoparticles on the expression of TXB2, 6-keto-PGF1, and TF in cerebral thrombosis model rats. **(A)** Expression of TBX2 in cerebral thrombosis model rats. **(B)** Expression of 6-keto-PGF1 in rats with cerebral thrombosis. **(C)** Expression of TF in rats with cerebral thrombosis. ^∗∗^*P* < 0.01 and ^∗∗∗^*P* < 0.001, compared with NC group; ^#^*P* < 0.05, ^##^*P* < 0.01, and ^###^*P* < 0.001, compared with Model group; ^@^*P* < 0.05, compared with APS group.

As shown in Figure 4, the expression of TXB2, 6-Keto-PGF1α, and TF in the Model group was significantly increased (*P* < 0.01) compared with the NC group, however, when compared with the Model group, the expressions of TXB2, 6-Keto-PGF1α, and TF in both APS-nano group and the APS group showed a downward trend (*P* < 0.05). Moreover, the expression of TXB2, 6-Keto-PGF1α, and TF in the APS-nano group was reduced (*P* < 0.05) compared with the APS group.

### Effects of Astragalus Polysaccharide and Its Nanoparticles on the Expression of NSE and S-100β in the Serum of Cerebral Thrombosis Model Rats

**FIGURE 5:**
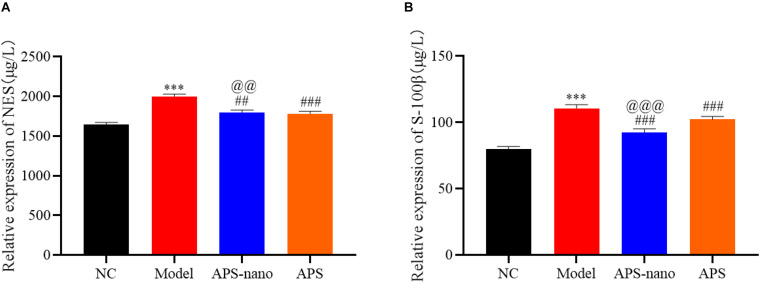
Effect of APS and APS nanoparticles on the expression of NSE and S-100β in the serum of cerebral thrombosis rats. **(A)** NES expression level in serum of cerebral thrombosis model rats. **(B)** S-100β expression level in serum of cerebral thrombosis model rats. ^∗∗∗^*P* < 0.001, compared with NC group; ^##^*P* < 0.01, and ^###^*P* < 0.001, compared with Model group; ^@@^*P* < 0.01 and ^@@@^*P* < 0.001, compared with APS group.

Compared with the NC group, the NSE and S-100β protein content in the Model group was increased significantly (*P* < 0.001). However, the content of NSE and S-100β protein in the APS-nano group and the APS group was decreased (*P* < 0.01) compared with the Model group. Similarly, the content of NSE and S-100β protein in the APS-nano group was decreased (*P* < 0.01) compared with the APS group, as shown in Figure 5.

### Effects of Astragalus Polysaccharide and Its Nanoparticles on the Content of CAT, SOD, and MDA in Brain Tissue of Cerebral Thrombosis Model Rats

**FIGURE 6:**
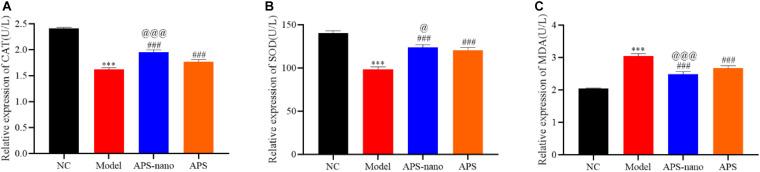
Effects of APS and APS nanoparticles on CAT, SOD, and MDA levels in cerebral thrombosis model rats. **(A)** Content of CAT in brain tissue of cerebral thrombosis model rats. **(B)** Content of SOD in brain tissue of cerebral thrombosis model rats. **(C)** Content of MDA in brain tissue of cerebral thrombosis model rats. ^∗∗∗^*P* < 0.001, compared with NC group; ^###^*P* < 0.001, compared with Model group; ^@^*P* < 0.05 and ^@@@^*P* < 0.001, compared with APS group.

As shown in Figure 6, the content of CAT and SOD in the Model group was significantly reduced, and the content of MDA was significantly increased (*P* < 0.001) compared with the NC group. However, the content of CAT and SOD in the APS-nano group and the APS group was increased, and the MDA content was decreased (*P* < 0.001) compared with the Model group. Similarly, the content of CAT and SOD in the APS-nano group was increased and the content of MDA was decreased (*P* < 0.05) compared with the APS group.

### APS-Nano Targets TF by Up-Regulating miRNA Expression

**FIGURE 7:**
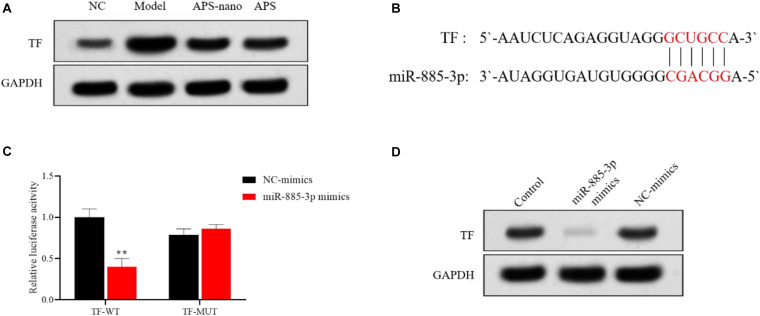
miR-885-3p targets the site of TF. **(A)** TF protein expression levels in each group. **(B)** Target binding sites of miR-885-3P and TF. **(C)** Experimental results of double luciferase reporter gene. **(D)** TF protein expression levels in each group. ^∗∗^*P* < 0.01, compared with the NC-mimics group.

Compared to the NC group, the expression of TF protein in the Model group was increased, but the TF protein in the APS-nano group and the APS group was decreased, as shown in Figure 7A. Predicted by the bioinformatics online analysis software miRBase, the results showed that TF may be the target gene of miR-885-3p, as shown in Figure 7B. Through the double luciferase report experiment, it was found that the luciferase activity of TF-WT in the miR-885-3p mimics group was significantly inhibited (*P* < 0.01), and the luciferase activity of TF-MUT did not change significantly, see Figure 7C. At the same time, compared with the Control group, the expression of TF protein in the miR-885-3p mimics group was significantly reduced, as shown in Figure 7D.

## Discussion

With the current changes in people’s lifestyles, the rate of cerebral thrombosis has increased year by year. Among them, hypertension, hyperlipidemia, atherosclerosis, and diabetes are the main causes of cerebral thrombosis. Since cerebral thrombosis often occurs in a quiet environment or sleep state, the treatment cannot be obtained in time, so it has the characteristics of poor postoperative recovery ability and a high disability rate (Balami et al., 2018). Studies have found that ADP can cause platelet aggregation, increased permeability of blood vessels and capillaries which leads to the destruction of the blood-brain barrier (Yan et al., 2018). Also, high expression of thrombin can cause fibrinogen in the blood to be converted into fibrin, which in turn can cause blood coagulation (Pleşeru and Mihailă, 2018). Furthermore, epinephrine causes vasoconstriction, which further activates platelet function, and promotes thrombosis (Kuiper, 2018). The compound inducer (ADP-thrombin-adrenaline) is used to induce rat cerebral thrombosis model, which can simulate the pathophysiological process of human cerebral thrombosis. It has the characteristics of convenient operation, short time, and high efficiency, and has been widely used in the investigation of the mechanism of cerebral thrombosis and the research of antithrombotic drugs. In the treatment of cerebral thrombosis, the curative effect of drugs is greatly reduced due to the existence of the blood-brain barrier. With the continuous deepening of research in recent years, it has been discovered that the nano-drug delivery system can cross the blood-brain barrier to reach the target site, thereby achieving the effectiveness of treating diseases.

Astragalus polysaccharide is the main bioactive substance of astragalus, which has pharmacological effects such as vascular protection, immune regulation, anti-inflammatory, and anti-fibrosis (Zeng et al., 2019). Studies have shown that Astragalus Decoction and Astragalus Injection can promote the treatment of stroke (Xu et al., 2017). The results of this study showed that compared with the NC group, rats in the Model group had severe cerebral thrombosis and cerebral edema, indicating that the rat brain thrombosis model was successfully constructed. When Astragalus polysaccharides were given for intervention, both the A/left brain wet weight ratio and the left brain/right brain wet weight ratio was decreased, that is, rat cerebral thrombosis and cerebral edema were significantly reduced, suggesting that astragalus polysaccharides can inhibit cerebral thrombosis in rats, reduce cerebral edema and inhibit the increase of cerebral vascular permeability. After the intervention of astragalus polysaccharide nanoparticles, the SD brain thrombosis model rats had lower A/left cerebral wet weight ratio and left brain/right brain wet weight ratio than astragalus polysaccharide intervention, which suggests that astragalus polysaccharide nanoparticles have better effects on the treatment of cerebral thrombosis.

The formation of cerebral thrombosis will result in obstacles in the body’s blood circulation system, leading to the slowing of blood flow velocity and the occurrence of eddy currents in local areas, causing abnormal coagulation function and blood rheology indicators (Schiano di Visconte et al., 2017). The results of this study showed that compared with the NC group, the whole blood low-cut viscosity, whole blood high-cut viscosity, plasma viscosity, and red blood cell aggregation index in the blood rheology index of the Model group were significantly increased, the proprotein content of fiber in the coagulation function test indicator was increased significantly and also the expression of TXB2 and 6-Keto-PGF1α were increased, but prothrombin time and activated partial thromboplastin time were decreased significantly, which suggested that the blood in the rat cerebral thrombosis model induced by the compound inducer was highly viscous. When given astragalus polysaccharides intervention treatment, it can make the model rat’s whole blood low-cut viscosity, whole blood high-cut viscosity, plasma viscosity, erythrocyte aggregation index gets a certain improvement. Also, after the intervention, the prothrombin time and activated partial thrombin time was increased, the fibrinogen content and the expression of TXB2 and 6-Keto-PGF1α were decreased. This is consistent with the results of blood flow changes during migraine attacks (Shayestagul et al., 2017). This shows that astragalus polysaccharide can improve the hemorheology and coagulation function of rat cerebral thrombosis model, and has a certain inhibitory effect on the formation and development of cerebral thrombosis. Compared with the astragalus polysaccharide intervention treatment, the astragalus polysaccharide nanoparticle intervention treatment has a better inhibitory effect on the cerebral thrombosis model rats.

Neuron-specific enolase (NSE) is a key step in the glycolysis pathway. It exists in the cytoplasm of neurons and neuroendocrine cells. When neurons are damaged or necrotic, NSE overflows from the cells, so it can be used to reflect the extent of neuronal damage in the acute phase of infarction (Arslan et al., 2020). S-100β protein is mainly present in astrocytes and glial cells of the central nervous system and it is a glial marker protein. When the brain is injured or the blood-brain barrier is destroyed, it can enter the blood, so it is a sensitive indicator reflecting the degree of brain damage and prognosis (Zhou et al., 2018). Compared with the NC group, the NSE and S-100β protein content in the Model group increased significantly. After astragalus polysaccharide intervention treatment, the content of NSE and S-100β protein in cerebral thrombosis model rats was reduced, and after astragalus polysaccharide nanoparticle intervention treatment, the trend of NSE and S-100β protein decline was more obvious. This shows that compared with Astragalus polysaccharides, astragalus polysaccharide nanoparticles can more effectively reduce the secondary damage of neurons and glial cells after cerebral thrombosis.

Superoxide dismutase and CAT are important indicators of oxidative and antioxidant systems in the human body. When this balance is broken, it will lead to increased membrane permeability, loss of function, mitochondrial dysfunction, lysosomal rupture, cell lysis, and tissue edema, increased permeability of blood vessels, and also promote the formation of cerebral edema and aggravate the condition (Gu et al., 2016). Compared with the NC group, the content of CAT and SOD in the Model group decreased significantly, and the content of MDA increased. Astragalus polysaccharide intervention treatment can increase the content of CAT and SOD and decrease the MDA content in rats with cerebral thrombosis model. Also, after the intervention treatment of astragalus polysaccharide nanoparticles, the content of CAT and SOD is much higher and the content of MDA is much lower. This shows that compared with Astragalus polysaccharides, astragalus polysaccharides nanoparticles can effectively enhance antioxidant enzyme activity, scavenge free radicals, and inhibit brain damage caused by lipid peroxidation.

Many studies showed that the specific surface area of the active ingredient of the Chinese medicine increased after coating the nano-carrier, which prolonged the drug’s *in vivo* retention time, in addition, it has the advantages of targeting and sustained release (Huang et al., 2015; Zhao et al., 2020). The astragalus polysaccharide nanoparticles prepared in this study had a smooth appearance, a longer release time *in vitro*, and a slow-release with a lower burst rate, which has the advantages of “nano traditional Chinese medicine”. *In vivo* studies have found that compared with Astragalus Polysaccharides, its nanoparticles showed better effects in the treatment of cerebral thrombosis. The above results indicate that Astragalus polysaccharide nanoparticles may achieve better clinical effects in the clinical treatment of cerebral thrombosis. However, most of the current research on nanoparticles is still in the drug preparation and animal experiment stage. The safety of its clinical application and its direct effects on humans is still unclear, which requires further study.

Tissue factor has important significance in thrombosis. Under normal conditions, the expression level of TF in plasma is low. When pathologically or physically stimulated, it will cause TF expression to increase and start the coagulation process, leading to the occurrence of thrombosis (Zhu et al., 2018). It has been found that TF is the main initiator of blood clotting in tumors, and silencing TF can inhibit blood-borne metastasis of tumors (Date et al., 2017). Compared with the Model group, the plasma TF content of cerebral thrombosis model rats decreased after astragalus polysaccharide intervention treatment, suggesting that astragalus polysaccharide may relieve cerebral thrombosis by reducing TF content. In this study, bioinformatics analysis showed that TF may be the target gene of miR-885-3p, and double luciferase experiments confirmed that the two can indeed directly bind, which is related to the formation of cerebral thrombosis, but it has not been discussed in this study and more research need to be done.

In summary, through research on the effects of thrombosis-related factors, blood rheology indicators, and blood coagulation function detection indicators, it was found that astragalus polysaccharides can play a certain role in relieving cerebral thrombosis, and astragalus polysaccharide nanoparticles have more significant inhibitory effects on cerebral thrombosis. Moreover, astragalus polysaccharide may play a role by miR-885-3p targeted regulation of TF expression. This study provides insights into the clinical treatment of cerebral thrombosis.

## Data Availability Statement

The original contributions presented in the study are included in the article/supplementary material, further inquiries can be directed to the corresponding author/s.

## Ethics Statement

The animal study was reviewed and approved by the Animal Care and Use Committee at Xinxiang Central Hospital.

## Author Contributions

QS and PS proposed and designed the experiments. CL and JM carried out the experiments with the help of QS. QS, PS, and CL contributed to data analysis, data interpretation, and manuscript preparations. All authors conceived and designed the study.

## Conflict of Interest

The authors declare that the research was conducted in the absence of any commercial or financial relationships that could be construed as a potential conflict of interest.
